# Improving Identification and Management of Modifiable Risk Factors in Hip and Knee Osteoarthritis: A Quality Improvement Project in an NHS Orthopaedic Interface Service

**DOI:** 10.1002/msc.70234

**Published:** 2026-05-18

**Authors:** Rose Henning, Michael Henning, Justine McMillan, Gethin Lynch

**Affiliations:** ^1^ Orthopaedic Interface Royal Devon University Healthcare NHS Foundation Trust Exeter UK; ^2^ Centre for Health and Clinical Research University of the West of England Bristol UK

## Abstract

**Background:**

Hip and knee osteoarthritis are highly prevalent conditions associated with pain, disability and increasing demand on orthopaedic services. Best practice guidance emphasises early identification and optimisation of modifiable risk factors to improve outcomes following joint replacement, yet these are often inconsistently addressed in routine musculoskeletal care. This quality improvement project aimed to improve the consistency with which clinicians identified and acted on key modifiable risk factors during orthopaedic interface consultations.

**Methods:**

The project was conducted in an NHS Orthopaedic Interface Service using the Model for Improvement. A retrospective audit of 291 consultations demonstrated low rates of documented action despite a high prevalence of modifiable risk factors. Four domains were prioritised: excess body weight, smoking, diabetes optimisation and psychosocial factors. A bundle of low‐burden interventions was introduced through sequential plan–do–study–act cycles. Prospective monthly sampling of 180 consultations over six months assessed change over time.

**Results:**

Across 180 prospective consultations, documented action increased from 30% to 78% among consultations where at least one targeted modifiable factor was present. Improvement was clearest for weight‐related advice and signposting. Smoking, diabetes and psychosocial findings were limited by small denominators or variable documentation and were interpreted descriptively. The largest increase was observed following introduction of embedded workflow supports, although attribution to any single component is limited by the sequential QI design.

**Conclusion:**

A simple quality improvement bundle was associated with improved documentation of action on modifiable risk factors during routine osteoarthritis consultations, particularly for weight‐related advice and signposting. Embedded documentation prompts may support more reliable care processes, but further work is needed to assess sustainability, patient‐level outcomes and transferability to other settings.

## 1 Background

Hip and knee osteoarthritis are major causes of pain, disability and reduced quality of life (Steinmetz et al. 2023). They account for a high volume of musculoskeletal consultations in primary care and are a significant contributor to elective orthopaedic waiting lists (Button et al. 2019). Best practice guidelines stress the importance of early identification of modifiable risk factors that influence symptoms, non‐operative management and perioperative outcomes (NHS England 2025). Advanced practice led interface services are well placed to support this process because they provide early access to senior clinical decision making and often act as a gateway to both conservative and surgical pathways (NHS England 2023, 2025). In the NHS, advanced practice refers to a level of practice characterised by high autonomy and complex decision making, typically working across the four pillars of clinical practice, leadership, education and research (NHS Employers 2023).

Several modifiable factors have clear links with osteoarthritis symptoms and with outcomes following joint replacement. Excess body weight, typically defined as a body mass index (BMI) ≥ 30 kg/m^2^, is associated with higher pain levels, reduced function and lower postoperative satisfaction (Chaudhry et al. 2019; Raud et al. 2020). Smoking is associated with delayed healing, postoperative complications and infection, and national guidance encourages clinicians to offer brief advice or signposting as part of routine musculoskeletal (MSK) care (Yue et al. 2022). Poor glycaemic control is associated with increased perioperative risk, particularly wound and infection outcomes, and is a priority for early optimisation in surgical pathways (Tarabichi et al. 2017). Psychosocial factors, including low mood, anxiety, and fear avoidance, are associated with greater pain, poorer coping and reduced engagement with rehabilitation (Fernández‐de‐Las‐Peñas et al. 2023). Addressing these factors early can support shared decision making and may improve the likelihood of success with both conservative management and surgical care (Lindner et al. 2018; NHS England 2025).

Despite this, a recent scoping review reported variability in how physiotherapists address weight, with uncertainty among clinicians and patients about whether this sits within the physiotherapy role (Steele‐Turner et al. 2025). Physiotherapists and students described limited confidence and skills in delivering weight‐related advice and signposting and noted that education and support in this area are often lacking. These findings mirrored barriers raised locally, where clinicians reported uncertainty about referral thresholds and difficulty incorporating lifestyle conversations into short appointments. Together, this highlights the need for simple and practical tools that help clinicians act on relevant modifiable factors during routine interface consultations.

Before the project began, clinicians within the Orthopaedic Interface Service reported that these factors were being identified and acted on inconsistently. Documentation was variable, and several clinicians described difficulty remembering local eligibility thresholds or finding appropriate signposting information within short appointments. These concerns were supported by the baseline audit, which showed that although BMI and smoking status were often recorded, the proportion of patients receiving relevant advice or referral was low. Discussions about diabetes optimisation were rare, and psychosocial factors were seldom described. These findings mirror national evidence showing that although lifestyle and modifiable risk factors are widely recognised as important, they are inconsistently identified and addressed in routine musculoskeletal care due to time pressure, competing priorities and uncertainty about clinical roles (Henning and Smith 2023; Steele‐Turner et al. 2025).

A baseline audit provided a snapshot of local practice and the rationale for structured improvement. It identified specific gaps, confirmed that clinicians valued the importance of these factors, and highlighted practical barriers that could be addressed with small changes. This created an opportunity to test whether a simple bundle of interventions could improve consistency in the identification and management of modifiable factors during routine osteoarthritis consultations.

## 2 Objectives

The project had two linked objectives.

1. To describe the characteristics of patients with hip and knee osteoarthritis seen within the Orthopaedic Interface Service and to compare the prevalence of key modifiable factors with published national data.
2. To improve the consistency with which clinicians identified and acted on relevant modifiable risk factors during routine consultations, using rapid quality improvement cycles supported by repeated measurement.

## 3 Aim

To increase the proportion of consultations in which clinicians identified and acted on at least one relevant modifiable risk factor for hip and knee osteoarthritis.

## 4 Methods

### 4.1 Study Design

This quality improvement project used the Model for Improvement, a structured framework for testing and implementing change in healthcare (Institute for Healthcare Improvement 2026a). The aim was to increase the proportion of consultations in which clinicians identified and acted on at least one relevant modifiable factor. Improvement was assessed using monthly process measures based on routine clinical documentation. Potential changes were generated from baseline audit findings, clinician feedback and behaviour change analysis, and were tested in small plan–do–study–act (PDSA) cycles, a structured iterative method for testing and refining changes in practice (Institute for Healthcare Improvement 2026b). Ethics review was not required because the work focused on service processes using anonymised records.

### 4.2 Setting

The project took place in an NHS Orthopaedic Interface Service, in South West England, serving a mixed urban and largely rural population. The service is clinically led by consultant physiotherapists, senior clinicians working at consultant level of practice, above advanced practice, combining expert clinical practice with leadership, education and service development roles (NHS Employers 2023), but who were not directly involved in the patient consultations included in this audit.

Patient consultations were undertaken by advanced practitioners who provided assessment, triage, shared decision‐making and pathway navigation for people with hip or knee osteoarthritis referred from primary care. Clinicians work autonomously within defined governance arrangements and review imaging, guide conservative management and determine when orthopaedic assessment is indicated.

### 4.3 Baseline Audit

A retrospective baseline audit was undertaken prior to the quality improvement phase to understand current practice and inform intervention design. The audit reviewed 291 consecutive consultations for hip or knee osteoarthritis in which the patient was referred on to Orthopaedics between January and June 2024. Data were extracted from routine electronic clinical records.

For each consultation, the presence of modifiable factors (weight, smoking status, diabetes, and psychosocial factors) and any documented action relating to these factors were recorded. Action was defined as documented advice, signposting, or referral.

The audit was used to identify gaps in current practice and to inform the selection of target behaviours and intervention components. Findings highlighted uncertainty around local eligibility thresholds for onward support, particularly for weight management services, inconsistent documentation, and difficulty accessing signposting information during short consultations. These insights directly informed the development of the clinician handout and the embedded electronic documentation prompt.

### 4.4 Selection of Modifiable Factors

To clarify which modifiable factors should be the focus of improvement work, the project team used a simple prioritisation matrix based on clinical importance, evidence base, relevance to osteoarthritis outcomes, and the degree of control clinicians had within a routine consultation. The prioritisation matrix used to select target domains is provided in Appendix 1. Weight, smoking, diabetes control and psychosocial factors scored highest across all domains. These areas were common in the local population, had well‐established associations with surgical and rehabilitation outcomes, and were amenable to brief intervention or signposting without requiring major pathway redesign. Other potential factors such as cardiovascular risk, frailty or wider social issues were considered but were judged to be either less directly linked to osteoarthritis outcomes or less feasible to address in the time available.

These four domains also aligned with local pathway priorities. Weight, smoking and diabetes feature in preoperative optimisation guidance used by anaesthetic and surgical teams. Psychosocial factors appear consistently in national osteoarthritis recommendations and have growing recognition in musculoskeletal literature. Together, they formed a clear and clinically relevant set of modifiable factors for targeted improvement within the service.

### 4.5 Intervention Development

Barriers identified in the baseline audit were mapped to the Capability, Opportunity, Motivation and Behaviour (COM‐B) model, which proposes that behaviour is shaped by the interaction of people's capability, opportunity and motivation (Michie et al. 2011). Capability barriers included uncertainty about referral thresholds for weight management, limited confidence in giving brief lifestyle advice and a lack of concise wording for documentation. These mirrored themes reported in wider literature on physiotherapy led osteoarthritis care, where clinicians describe uncertainty about whether weight and lifestyle discussions sit within their role (Steele‐Turner et al. 2025). Opportunity barriers relate to the absence of prompts or easy access to signposting information during consultations. Motivation barriers included competing priorities in short appointments and low expectation that lifestyle discussions would influence clinical outcomes. The intervention bundle was designed to target these domains.

The clinician handout addressed capability by clarifying thresholds and local options. Opportunity was improved through a visible desk resource and later through an EPIC SmartPhrase, a predefined electronic documentation template that inserts standardised text and signposting links at the point of care. Motivation was supported by framing lifestyle discussions as part of core osteoarthritis care and by providing simple wording for documentation.

A pre‐existing musculoskeletal Patient and Public Involvement and Engagement (PPIE) group, convened for a separate research programme, had previously discussed preferences for brief, practical lifestyle messaging. These informal insights helped shape the format and tone of the materials, although the group did not take part in formal co‐design or delivery of the intervention.

### 4.6 Improvement Strategy

Three PDSA cycles were carried out from late September to late November 2025.

#### 4.6.1 PDSA Cycle 1. Audit Feedback and Shared Learning (Late September 2025)

Plan: Present audit findings and agree on priority behaviours.

Do: Findings were shared in a team meeting. Clinicians highlighted inconsistent documentation and uncertainty about referral thresholds.

Study: Clinicians reported that a simple reference guide would help address these gaps.

Act: The team agreed to develop a concise handout for testing.

#### 4.6.2 PDSA Cycle 2. Clinician Handout (Early November 2025)

Plan: Create and test a quick reference sheet covering thresholds and local options.

Do: A one page guide was placed in clinic rooms for clinicians to use.

Study: Feedback indicated that it improved confidence, but clinicians still spent time locating suitable wording for documentation.

Act: The next cycle focused on creating a SmartPhrase to streamline documentation.

#### 4.6.3 PDSA Cycle 3. EPIC SmartPhrase (Late November 2025)

Plan: Develop a SmartPhrase with standardised text and links.

Do: Clinicians tested the SmartPhrase in routine consultations over 3 weeks.

Study: Clinicians reported faster documentation, clearer signposting and more consistent language.

Act: Minor refinements were made and the SmartPhrase was adopted for ongoing use.

## 5 Outcome Measures

### 5.1 Primary Process Measure

The primary process measure was the proportion of applicable consultations in which clinicians documented action on at least one relevant modifiable factor. An applicable consultation was defined as a consultation where at least one targeted modifiable factor was present. Acting on a factor included documented advice, signposting or referral. The denominator for the primary process measure was therefore the number of applicable consultations each month, rather than the total number of consultations reviewed.

### 5.2 Secondary Process Measures

1. Proportion of patients with excess body weight, operationalised as body mass index (BMI) ≥ 30 kg/m^2^ in line with local eligibility criteria for structured weight management services, who received weight‐related advice or signposting.
2. Proportion of current smokers who received smoking cessation advice or signposting.
3. Proportion of patients with diabetes in whom glycaemic control was documented, and where optimisation was required, the proportion with documented discussion or onward action.

### 5.3 Operational Definitions

For the purposes of this project, an applicable consultation was operationally defined as any consultation in which at least one targeted modifiable risk factor was present. The presence of a factor was determined from documented clinical history or routinely recorded data. This definition was developed to ensure that process measures reflected opportunities where action was clinically relevant.

Action on a modifiable factor was defined as any documented advice, signposting, or referral relating to that factor during the consultation. Actions did not need to result in referral acceptance or behaviour change to be counted.

The primary process measure assessed whether any relevant modifiable factor was acted on during an applicable consultation. Factor‐specific secondary measures used narrower denominators based on the presence of each individual factor, for example BMI ≥ 30 kg/m^2^, current smoking or diabetes.

For weight management, applicable consultations were those where body mass index (BMI) was 30 kg/m^2^ or above, in line with local eligibility criteria for structured weight management services. Action included brief advice, signposting to digital or Tier 2 weight management programmes, or referral where appropriate.

For smoking, applicable consultations were those in which the patient was documented as a current smoker. Action included brief cessation advice, signposting to smoking cessation services, or referral.

For diabetes, two related behaviours were assessed descriptively: first, documentation of glycaemic status, defined as HbA1c being recorded for patients with a diagnosis of diabetes and second, where HbA1c was ≥ 69 mmol/mol, documentation of discussion, signposting or communication with primary care regarding optimisation. Because the number of patients with diabetes was small, these components were not interpreted as evidence of improvement over time.

Psychosocial factors were recorded descriptively. Documentation of psychosocial factors was defined as any explicit reference in the clinical record to mood disturbance, anxiety, depression, sleep problems, fear avoidance, coping difficulties or psychosocial stressors. Psychosocial factors were not included as a formal process measure because the intervention did not directly target psychosocial assessment or screening.

### 5.4 Data Collection

Prospective data collection was undertaken from August 2025 to January 2026. Each month, 30 consecutive new consultations for hip or knee osteoarthritis were reviewed using routinely recorded electronic clinical records. Sampling was distributed across clinicians to minimise individual practice effects.

August and September 2025 served as the prospective baseline period for the run chart because these data were collected using the same monthly sampling approach as the post‐intervention period. The earlier retrospective audit was used to characterise local need and inform intervention design, but was not used as the run chart baseline because it differed in timing, purpose and sampling approach. This decision improved consistency within the prospective quality improvement evaluation, although it reduced the number of baseline data points available for run chart interpretation.

For each consultation, the presence of targeted modifiable risk factors and whether documented action occurred were coded using pre‐specified operational definitions.

### 5.5 Analysis

Monthly proportions for the primary process measure were plotted on a run chart with intervention points annotated. A baseline median was calculated using August and September because these were the two prospectively collected pre‐intervention months. Run chart interpretation considered standard rules for non‐random variation, including evidence of a shift or trend. Given the short two‐month prospective baseline and limited number of monthly data points, run chart findings were interpreted cautiously and were not treated as definitive evidence of special cause variation. Secondary measures were summarised descriptively. Data extraction, data management and descriptive analysis were undertaken in Microsoft Excel. No hypothesis testing was undertaken because the purpose was quality improvement rather than formal inference.

### 5.6 Reporting Guideline

This project is reported in line with the SQUIRE 2.0 reporting guideline for quality improvement studies (Ogrinc et al. 2016).

### 5.7 Ethics

This project was conducted as a quality improvement initiative and did not require formal research ethics committee approval. The work was registered with the NHS Trust audit and service evaluation department and was conducted using anonymised routinely collected clinical data.

## 6 Results

### 6.1 Baseline Patient Characteristics

The baseline audit included 291 consultations for hip and knee osteoarthritis. Mean BMI was 30.9 kg/m^2^. Overall, 86% of patients were overweight and 51% were obese. Diabetes was present in 16%, hypertension in 49%, cardiovascular disease in 24%, and 5% were current smokers. Psychosocial factors such as sleep or mood disturbance were infrequently documented. Table 1 summarises patient characteristics and modifiable risk factors compared with national data. A comparison with national epidemiological data is provided in Table 1.

**TABLE 1:** Comparison of patient characteristics and modifiable risk factors.

Factor	Local audit	National average^a^
BMI (mean)	30.9	27.8
Overweight (BMI > 25)	86%	66%
Obese (BMI > 30)	51%	27%
Diabetes	16%	9%
Hypertension	49%	30%
Cardiovascular disease	24%	13%
Current smoker	5%	11%

^a^ National estimates derived from NHS Digital data for England (NHS Digital 2026), based on adult population data.

These findings confirmed that a large proportion of patients had modifiable risk factors with clear clinical relevance, yet action was documented in only 11% of consultations. This highlighted a gap between clinical need and practice and informed the focus of the improvement work.

### 6.2 Primary Process Measure

The retrospective audit showed that only 11% of consultations documented action on a modifiable factor. These data were used to identify the improvement opportunity and inform about intervention design but were not included in the run chart baseline because they were collected using a different retrospective sampling approach.

Six months of prospective sampling from August 2025 to January 2026 yielded 180 consultations, with 30 consultations reviewed each month. The denominator for the primary process measure was the number of applicable consultations each month, defined as consultations where at least one targeted modifiable factor was present. Across the six months, 90 of 180 consultations were applicable to the primary process measure. Monthly numerators, denominators and percentages for the primary process measure are shown in Table 2.

**TABLE 2:** Primary process measures during the prospective quality improvement period.

Month	Consultations reviewed	Applicable consultations	Consultations with documented action	Primary process measure (%)
August 2025	30	10	3	30
September 2025	30	18	7	39
October 2025	30	16	6	38
November 2025	30	15	10	67
December 2025	30	13	9	69
January 2026	30	18	14	78
**Total**	**180**	**90**	**49**	**54**

*Note:* The bold values indicate the overall totals across the six‐month prospective quality improvement period. The values 180, 90 and 49 are summed totals for consultations reviewed, applicable consultations and consultations with documented action. The final bold value, 54%, is the overall primary process measure across the full period, calculated as 49/90, rather than an average of the monthly percentages.

In August and September, which served as the prospective baseline for the run chart, clinicians documented action on a relevant modifiable factor in 30% (3/10) and 39% (7/18) of applicable consultations. Following the first PDSA cycle in early October, this was 38% (6/16). After introduction of the clinician handout in November, this increased to 67% (10/15). Following introduction of the SmartPhrase in late November, the rate was 69% (9/13) in December and 78% (14/18) in January 2026.

**FIGURE 1:**
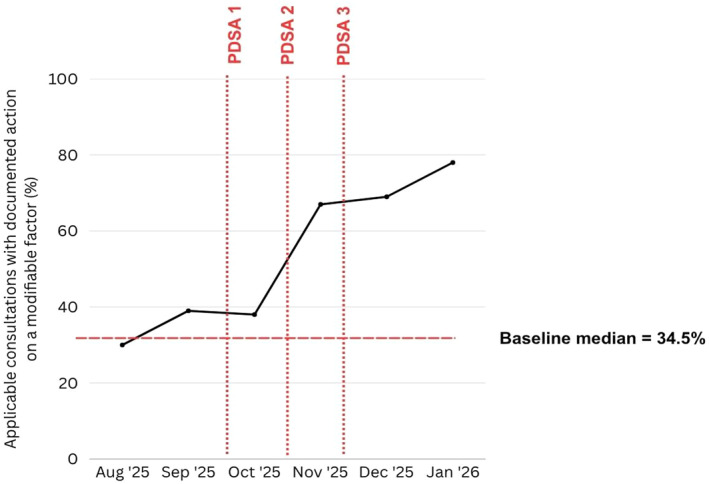
Monthly proportion of applicable consultations in which clinicians documented action on at least one relevant modifiable factor from August 2025 to January 2026. The dashed horizontal line represents the median calculated from the two prospectively collected baseline months, August and September 2025. The earlier retrospective audit was not included in the run chart baseline. PDSA intervention points are annotated.

The baseline median calculated from August and September was 34.5%. Visual inspection suggested improvement over time, with all points from September 2025 to January 2026 above the baseline median. However, the run chart did not meet the conventional rule for a formal shift, which requires six or more consecutive points on one side of the median, nor did it meet the criteria for a formal trend. The run chart was therefore interpreted as showing improvement over time in documented action, but not definitive evidence of special cause variation. Figure 1 shows the monthly primary process measure with PDSA intervention points annotated.

### 6.3 Secondary Process Measures

Factor‐specific secondary outcomes were interpreted cautiously. Weight‐related advice and signposting had sufficient prevalence for month‐by‐month description, whereas smoking, diabetes and psychosocial factors had small denominators or variable documentation and were therefore reported as pooled descriptive counts.

#### 6.3.1 Weight Management

Weight‐related advice and signposting showed the clearest factor‐specific improvement. Among patients with BMI ≥ 30 kg/m^2^, documented advice or signposting increased from 25% (2/8) in August to 86% (12/14) in January 2026. Month‐by‐month denominators and percentages are shown in Table 3.

**TABLE 3:** Weight‐related advice and signposting among patients with BMI ≥ 30 kg/m^2^ during the prospective quality improvement period.

Month	Consultations reviewed	Patients with BMI ≥ 30 kg/m^2^	Patients with BMI ≥ 30 kg/m^2^ receiving documented advice or signposting (%)
August 2025	30	8	2/8, 25
September 2025	30	12	6/12, 50
October 2025	30	15	6/15, 40
November 2025	30	10	8/10, 80
December 2025	30	12	8/12, 67
January 2026	30	14	12/14, 86

#### 6.3.2 Smoking

Current smokers represented a small proportion of consultations across the project period. Smoking status was recorded in all 180 consultations, with 11 patients identified as current smokers. Smoking cessation advice or signposting was documented in 6 of these 11 cases. Because monthly denominators ranged from 0 to 3 smokers, smoking outcomes were reported as pooled descriptive counts rather than month‐by‐month percentages and were not interpreted as evidence of change over time.

#### 6.3.3 Diabetes

Thirteen patients had a diagnosis of diabetes during the six‐month period. HbA1c was documented in five cases. One patient had an HbA1c above the optimisation threshold, at 82 mmol/mol, and in this case both signposting to patient resources and onward communication with the general practitioner were documented. Because monthly denominators ranged from 1 to 3 patients with diabetes, diabetes outcomes were reported descriptively and were not interpreted as evidence of change over time.

#### 6.3.4 Psychosocial

Psychosocial issues were documented infrequently throughout the project period. Across the six‐month audit period, psychosocial factors were documented in 9 consultations, with signposting to psychosocial support recorded in 2 cases. Because psychosocial assessment was not directly targeted by a structured prompt, screening tool or defined intervention component, these findings should be interpreted as descriptive evidence of limited documentation rather than as an evaluated psychosocial outcome.

## 7 Discussion

This quality improvement project was associated with improved documentation of action on modifiable risk factors during routine hip and knee osteoarthritis consultations in an NHS Orthopaedic Interface Service. Baseline audit findings showed that modifiable factors were common but infrequently acted on. During the prospective quality improvement period, documented action increased among applicable consultations, particularly for weight‐related advice and signposting. This improvement reflects increased consistency of documented action rather than direct evidence of patient behaviour change or engagement with optimisation services or clinical outcomes. The project was designed to improve process reliability at the point of care, and assessing downstream effects such as engagement with services, metabolic control or surgical outcomes was beyond its scope.

The timing of improvement suggests that embedded workflow supports may have contributed to more reliable documentation, but causal attribution to the SmartPhrase alone is limited. The interventions were introduced sequentially and may have had cumulative effects, including audit feedback, team discussion, the clinician handout and later the SmartPhrase. In addition, secular changes, repeated measurement and increasing clinician awareness may have influenced documentation over time. The SmartPhrase appeared useful because it placed standardised wording and signposting options within the note‐writing workflow, reducing reliance on memory during time‐pressured consultations. This is consistent with the COM‐B model, where increasing opportunity and reducing friction can support behaviour change. However, the findings should be interpreted as an association between the QI bundle and improved documentation, rather than as evidence that any single component independently caused the observed improvement.

Factor‐specific findings were strongest for weight‐related advice and signposting. This was clinically relevant because the retrospective audit highlighted a high prevalence of excess body weight in this interface population, with 86% of patients overweight and 51% living with obesity. The clearer improvement in this domain may also reflect the high prevalence of BMI ≥ 30 kg/m^2^ within the prospective samples, clear local eligibility thresholds and established signposting options. In contrast, smoking and diabetes were present in small numbers within the monthly samples, limiting interpretation of change over time. These outcomes are therefore best viewed as descriptive signals rather than evidence of improvement or lack of improvement. Diabetes documentation also highlighted an operational issue: although HbA1c‐related action was documented when a value was clearly above the optimisation threshold, routine recording of HbA1c for all patients with diabetes was less consistently embedded. Future iterations may benefit from clearer expectations, prompts to record HbA1c status, and closer links with perioperative or diabetes specialist nursing teams.

Psychosocial factors were clinically relevant to the project but were operationally weaker than the other domains. Although psychosocial support options were included within the signposting resources, no structured screening question, prompt or defined assessment process was introduced. Low documentation of psychosocial factors should therefore not be interpreted as evidence that psychosocial issues were absent or unchanged. Instead, it highlights a residual implementation gap: brief signposting resources alone may be insufficient to support consistent psychosocial assessment or action in routine interface consultations.

For clinicians and services, the main practical message is that concise prompts, standardised wording and accessible signposting information may support more reliable documentation of modifiable risk factor discussions. For researchers and service leads, the next step is to evaluate whether improved process reliability is sustained and whether it translates into patient engagement with support services, improved health optimisation or better downstream outcomes. Future work should also assess transferability to other musculoskeletal and advanced practice settings.

### 7.1 Limitations and Lessons Learnt

This project was conducted within a single orthopaedic interface service, which may limit generalisability. The evaluation relied on clinical documentation, and discussions that occurred but were not recorded would not have been captured. The prospective follow‐up period was shorter and patient level outcomes such as weight change, smoking cessation, improvements in glycaemic control, symptoms, engagement with support services or surgical readiness, were not measured. The project therefore demonstrates improved reliability of care processes at the point of consultation, rather than evidence of downstream clinical impact. As a result, it cannot be determined whether these changes translated into meaningful improvements in patient health or surgical outcomes.

Run chart interpretation was limited by the short two‐month prospective baseline and the small number of monthly data points. Although the chart showed improvement over time, it did not meet the standard rules for a formal shift or trend. Continued measurement over a longer period would be needed to determine whether improvement was sustained and whether special cause variation could be demonstrated more robustly.

Factor‐specific measures were constrained by small denominators. Smoking and diabetes were present in few consultations in each monthly sample, which limited sensitivity to detect change and reduced the usefulness of month‐by‐month percentages. Psychosocial factors were also limited by variable documentation and the absence of a structured assessment or screening component. These domains should therefore be interpreted descriptively and not as evidence of meaningful improvement or lack of improvement.

A further learning point was the ambiguity in how diabetes was operationalised during the early phases of the work. While the audit criteria assessed whether HbA1c status was documented for all patients with diabetes and whether cases above the optimisation threshold prompted discussion or action, this expectation was not explicitly communicated to clinicians. In practice, documentation and action tended to focus on patients with clearly elevated HbA1c. Future iterations would benefit from clearer operational definitions, explicit communication of documentation expectations, and prompts that support recording HbA1c status even when optimisation is not required.

Psychosocial factors were included within the signposting resources but were not supported by a structured assessment prompt or a screening process. This limits interpretation of psychosocial findings and means that the project cannot determine whether psychosocial assessment or management improved. Future work should consider whether structured prompts, brief screening questions or consultation support tools can improve recognition and documentation of psychosocial factors in routine interface consultations. Balance measures such as consultation duration and patient experience were not formally assessed and should also be considered in future work.

## 8 Conclusion

This quality improvement project found that a simple, locally designed intervention bundle was associated with improved documentation of action on modifiable risk factors during routine hip and knee osteoarthritis consultations. Improvement was clearest for weight‐related advice and signposting. Findings for smoking, diabetes and psychosocial factors were limited by small denominators or weaker operationalisation and should be interpreted descriptively. Embedded workflow supports, including standardised electronic documentation prompts, may help improve the reliability of care processes, but further evaluation is needed to assess sustainability, patient‐level outcomes and transferability to other settings.

## Author Contributions

**Rose Henning:** conceptualization, methodology, investigation, data curation, project administration, writing – review and editing. **Michael Henning:** conceptualization, methodology, formal analysis, data curation, writing – original draft, writing – review and editing. **Justine McMillan:** conceptualization, investigation, project administration, writing – review and editing. **Gethin Lynch:** conceptualization, methodology, supervision, writing – review and editing.

## Funding

The authors have nothing to report.

## Ethics Statement

This project was conducted as a quality improvement initiative and did not require formal research ethics committee approval. The work was registered with the NHS Trust audit and service evaluation department and was conducted using anonymised routinely collected clinical data.

## Conflicts of Interest

M.H. is supported by a National Institute for Health and Care Research (NIHR) Pre‐doctoral Clinical and Practitioner Academic Fellowship and holds an academic affiliation with the University of the West of England. This funding and affiliation did not support or influence the design, conduct, analysis or reporting of this project. No other conflicts of interest are declared.

## Data Availability

The data that support the findings of this study are available from the corresponding author upon reasonable request.

## Appendix 1 Prioritisation Matrix Used to Select Target Modifiable Factors

Target domains for improvement were selected using a simple project team prioritisation matrix. Candidate factors were scored across four domains:

1. Clinical importance
2. Strength of evidence linking the factor to osteoarthritis symptoms, rehabilitation or surgical outcomes
3. Relevance to routine orthopaedic interface consultations
4. Feasibility of clinician action within a standard consultation

Each domain was scored from 1 to 3, where 1 = low, 2 = moderate, and 3 = high. Total scores were used to guide the selection of factors for inclusion in the intervention bundle.

**Table:**

Factor	Clinical importance	Evidence base	Relevance to OA outcomes	Feasibility of clinician action in routine consultation	Total
Excess body weight	3	3	3	3	12
Smoking	3	3	3	2	11
Diabetes optimisation	3	3	3	2	11
Psychosocial factors	3	3	3	2	11
Cardiovascular risk factors	2	2	2	1	7
Frailty	2	2	2	1	7
Wider social factors	3	2	2	1	8

*Note:* Excess body weight, smoking, diabetes optimisation and psychosocial factors were prioritised for improvement because they scored highest overall and were considered both clinically relevant and feasible to address through brief advice, signposting or referral during routine consultations.

## References

[msc70234-bib-0001] Button, K. , F. Morgan , A. L. Weightman , and S. Jones . 2019. “Musculoskeletal Care Pathways for Adults With Hip and Knee Pain Referred for Specialist Opinion: A Systematic Review.” BMJ Open 9, no. 9: e027874. 10.1136/bmjopen-2018-027874. PMC6731906 31488471

[msc70234-bib-0002] Chaudhry, H. , K. Ponnusamy , L. Somerville , R. W. McCalden , J. Marsh , and E. M. Vasarhelyi . 2019. “Revision Rates and Functional Outcomes Among Severely, Morbidly, and Super‐Obese Patients Following Primary Total Knee Arthroplasty: A Systematic Review and Meta‐Analysis.” JBJS Reviews 7, no. 7: e9. 10.2106/jbjs.rvw.18.00184. 31365448

[msc70234-bib-0003] Fernández‐de‐Las‐Peñas, C. , L. L. Florencio , A. I. de‐la‐Llave‐Rincón , et al. 2023. “Prognostic Factors for Postoperative Chronic Pain After Knee or Hip Replacement in Patients With Knee or Hip Osteoarthritis: An Umbrella Review.” Journal of Clinical Medicine 12, no. 20: 6624. 10.3390/jcm12206624. 37892762 PMC10607727

[msc70234-bib-0004] Henning, M. , and M. Smith . 2023. “The Ability of Physiotherapists to Identify Psychosocial Factors in Patients With Musculoskeletal Pain: A Scoping Review.” Musculoskeletal Care 21, no. 2: 502–515. 10.1002/msc.1725. 36564962

[msc70234-bib-0005] Institute for Healthcare Improvement . 2026a. “Model for Improvement.” Model for Improvement. https://www.ihi.org/library/model‐for‐improvement.

[msc70234-bib-0006] Institute for Healthcare Improvement . 2026b. Plan‐Do‐Study‐Act (PDSA) Worksheet. https://www.ihi.org/library/tools/plan‐do‐study‐act‐pdsa‐worksheet.

[msc70234-bib-0007] Lindner, M. , O. Nosseir , A. Keller‐Pliessnig , P. Teigelack , M. Teufel , and S. Tagay . 2018. “Psychosocial Predictors for Outcome After Total Joint Arthroplasty: A Prospective Comparison of Hip and Knee Arthroplasty.” BMC Musculoskeletal Disorders 19, no. 1: 159. 10.1186/s12891-018-2058-y. 29788969 PMC5964720

[msc70234-bib-0008] Michie, S. , M. van Stralen , and R. West . 2011. “The Behaviour Change Wheel: A New Method for Characterising and Designing Behaviour Change Interventions.” Implementation Science 6, no. 1: 42. 10.1186/1748-5908-6-42. 21513547 PMC3096582

[msc70234-bib-0009] NHS England . 2025. NHS England, Early Screening, Triaging, Risk Assessment and Health Optimisation in Perioperative Pathways: Guide for Providers and Integrated Care Boards. https://www.england.nhs.uk/long‐read/earlier‐screening‐risk‐assessment‐and‐health‐optimisation‐in‐perioperative‐pathways/.

[msc70234-bib-0010] NHS Digital . 2026. “Health Survey for England, 2024.” Health Survey for England, 2024. https://digital.nhs.uk/data‐and‐information/publications/statistical/health‐survey‐for‐england/2024.

[msc70234-bib-0011] NHS Employers . 2023. Advanced Practice, Advanced Practice. https://www.nhsemployers.org/articles/advanced‐practice.

[msc70234-bib-0012] NHS England . 2023. Musculoskeletal Orthopaedic Approach to Referral Optimisation. https://www.england.nhs.uk/long‐read/msk‐orthopaedic‐approach‐to‐referral‐optimisation/.

[msc70234-bib-0013] Ogrinc, G. , L. Davies , D. Goodman , P. Batalden , F. Davidoff , and D. Stevens . 2016. “SQUIRE 2.0 (Standards for Quality Improvement Reporting Excellence): Revised Publication Guidelines From a Detailed Consensus Process.” BMJ Quality and Safety 25, no. 12: 986–992. 10.1136/bmjqs-2015-004411. PMC5256233 26369893

[msc70234-bib-0014] Raud, B. , C. Gay , C. Guiguet‐Auclair , et al. 2020. “Level of Obesity Is Directly Associated With the Clinical and Functional Consequences of Knee Osteoarthritis.” Scientific Reports 10, no. 1: 3601. 10.1038/s41598-020-60587-1. 32107449 PMC7046749

[msc70234-bib-0015] Steele‐Turner, B. , A. Gonçalves , A. Shepherd , et al. 2025. “Physiotherapist‐Led Weight Management for People With Osteoarthritis: A Scoping Review.” Osteoarthritis and Cartilage 34, no. 1: S1063458425012373. 10.1016/j.joca.2025.11.009. 41276008

[msc70234-bib-0016] Steinmetz, J. D. , G. T. Culbreth , L. M. Haile , et al. 2023. “Global, Regional, and National Burden of Osteoarthritis, 1990–2020 and Projections to 2050: A Systematic Analysis for the Global Burden of Disease Study 2021.” Lancet Rheumatology 5, no. 9: 508–522. 10.1016/s2665-9913(23)00163-7. PMC10477960 37675071

[msc70234-bib-0017] Tarabichi, M. , N. Shohat , M. M. Kheir , et al. 2017. “Determining the Threshold for HbA1c as a Predictor for Adverse Outcomes After Total Joint Arthroplasty: A Multicenter, Retrospective Study.”Supplement, Journal of Arthroplasty 32, no. 9: S263–S267. 10.1016/j.arth.2017.04.065. 28662955

[msc70234-bib-0018] Yue, C. , G. Cui , M. Ma , et al. 2022. “Associations Between Smoking and Clinical Outcomes After Total Hip and Knee Arthroplasty: A Systematic Review and Meta‐Analysis.” Frontiers in Surgery 9: 970537. 10.3389/fsurg.2022.970537. 36406352 PMC9666709

